# Use of Portable ECG 6Lead device compared to 12 Lead ECG for QTc monitoring of patients during initiation and monitoring of anti-psychotic medication. Identifying the preferences and views of service users and staff with time and cost efficiencies.

**DOI:** 10.1192/j.eurpsy.2026.12061

**Published:** 2026-07-31

**Authors:** L. Bennett, M. S. Krishnan

**Affiliations:** 1 Research and Development, Tees, Esk and Wear Valleys NHS Foundation Trust, Middlesbrough; 2 Old Age Psychiatry, Tees, Esk and Wear Valleys NHS Foundation Trust, Stockton, United Kingdom

## Abstract

**Introduction:**

Antipsychotic medications are associated with cardiac risks necessitating routine ECG monitoring, as recommended by national and international guidelines. Traditional 12-lead ECGs often increase patient anxiety and procedural burden in mental health settings. Recent technological advancements have introduced portable 6-lead ECG devices with instant QTc calculation. However, their acceptability and clinical utility in psychiatric practice remain underexplored.

**Objectives:**

To evaluate the acceptability, user experience, and efficiency of the KardiaMobile 6-lead ECG device with instant QTc measurement, compared with both manual QTc 6-lead and standard 12-lead ECGs, among mental health service users and clinical staff during antipsychotic medication initiation and monitoring.

**Methods:**

This real-world evaluation involved 161 service users and six mental health teams. Participants underwent ECG monitoring with 6-lead device with instant QTc calculation and traditional 12-lead ECG. Preferences, satisfaction, and experiences regarding privacy, dignity, invasiveness, and accommodation of personal beliefs were recorded. Staff preferences, procedural times, and cost implications were analysed.

**Results:**

The majority of service users (86.6%) and staff (89%) preferred the 6-lead device with instant QTc calculation. Service users reported reduced anxiety and high satisfaction regarding privacy and dignity. Implementation of the instant QTc 6-lead device was associated with streamlined workflows, faster clinical decision-making, and improved patient and staff experiences. Cost and time analyses suggested improved efficiency compared to traditional ECG approaches.

**Conclusions:**

The KardiaMobile 6-lead ECG device with instant QTc calculation demonstrates high acceptability and operational efficiency in mental health settings, supporting its integration into antipsychotic medication pathways. Wider adoption of portable ECG technology may enhance person-centred, clinically effective, and resource-efficient care in psychiatry.

**Disclosure of Interest:**

None Declared

